# DYT-40, a novel synthetic 2-styryl-5-nitroimidazole derivative, blocks malignant glioblastoma growth and invasion by inhibiting AEG-1 and NF-κB signaling pathways

**DOI:** 10.1038/srep27331

**Published:** 2016-06-02

**Authors:** Meijuan Zou, Yongtao Duan, Pengfei Wang, Rui Gao, Xuguan Chen, Yingwei Ou, Mingxing Liang, Zhongchang Wang, Yi Yuan, Li Wang, Hailiang Zhu

**Affiliations:** 1 Department of Pharmacology, School of Basic Medical Sciences, Nanjing Medical University, Nanjing 210029, China; 2 State Key Laboratory of Pharmaceutical Biotechnology, Nanjing University, Nanjing 210093, China; 3 Jiangsu Key Laboratory of Oral Diseases; Department of oral and maxillofacial surgery, Affiliated Hospital of Stomatology, Nanjing Medical University, Nanjing 210029, China; 4 Department of Breast Surgery, First Affiliated Hospital of Nanjing Medical University, Nanjing 210029, China

## Abstract

Astrocyte elevated gene-1 (AEG-1) has been explored as a novel target for human glioma therapy, thus reflecting its potential contribution to gliomagenesis. In the present study, we investigated the effect of DYT-40, a novel synthetic 2-styryl-5-nitroimidazole derivative, on cell growth and invasion in glioblastoma (GBM) and uncovered the underlying mechanisms of this molecule. DYT-40 induces the intrinsic mitochondrial pathway of apoptosis and inhibits the epithelial-mesenchymal transition (EMT) and invasion of GBM cell lines. Furthermore, DYT-40 deactivates PI3K/Akt and MAPK pathways, suppresses AEG-1 expression, and inhibits NF-κB nuclear translocation. DYT-40 reduced the tumor volumes in a rat C6 glioma model by apoptotic induction. Moreover, HE staining demonstrated that the glioma rat model treated with DYT-40 exhibited better defined tumor margins and fewer invasive cells to the contralateral striatum compared with the vehicle control and temozolomide-treated rats. Microscopic examination showed a decrease in AEG-1-positive cells in DYT-40-treated rats compared with the untreated controls. DYT-40-treatment increases the *in vivo* apoptotic response of glioma cells to DYT-40 treatment by TUNEL staining. In conclusion, the inhibitory effects of DYT-40 on growth and invasion in GBM suggest that DYT-40 might be a potential AEG-1 inhibitor to prevent the growth and motility of malignant glioma.

Malignant gliomas, such as glioblastoma multiforme (GBM) and anaplastic astrocytomas (AA), are the most common primary brain tumors^1^. GBMs are aggressive and destructive tumors that attack cerebral hemispheres with high frequency^2^. GBMs rapidly invade the surrounding brain parenchyma and contribute to gliomagenesis and resistance to traditional therapies^3^. Although multimodal therapies such as surgery, chemotherapy and radiation have been applied, the median survival of patients with GBMs is only 12–15 months^4^. GBMs continuously develop because the multiple genetic alterations accumulate step by step, followed by the activation of oncogenes and the inactivation of tumor suppressor genes. Thus, to determine a more effective targeted therapy for GBMs, the molecules critical for glioma progression should be determined, and the most effective inhibitors against these carcinogenic molecules should be identified.

Astrocyte elevated gene-1 (AEG-1, also known as MTDH, Lyric/3D3), a novel HIV-1- and TNF-α-inducible gene in primary human fetal astrocytes (PHFA), was originally cloned in Fisher’s laboratory^5^,^6^. Previous studies have characterized the mechanism of Ha-ras-mediated tumorigenesis and delineated the crucial role of AEG-1 in promoting cancer development and maintenance^7^. The over-expression of AEG-1 enhances the anchorage-independent growth and invasion of human cervical cancer, malignant glioma, prostate cancer, neuroblastoma, and hepatocellular carcinoma cells^8^,^9^,^10^,^11^. In contrast, the knockdown of AEG-1 expression significantly inhibits these phenotypes in malignant glioma and neuroblastoma^11^,^12^. Previous studies have demonstrated that the ectopic over-expression of AEG-1 promoted epithelial-mesenchymal transition (EMT), which resulted from the down-regulation of E-cadherin and the up-regulation of vimentin in lung cancer cell lines and clinical lung cancer specimens^13^. In these contexts, AEG-1 might provide a viable target for clinical therapeutic intervention in the EMT-mediated invasion of carcinomas.

Ras activation initiates a complex axis of transduction, including the Raf/MAPK (ERK) pathway, originally involved in the plasma membrane-to-nucleus signaling crucial for cell mitogen-mediated proliferation^14^ and the phosphatidylinositol 3-kinase (PI3K) Akt pathway, which is involved in cell survival signaling^15^. Akt stabilizes C-myc via phosphorylation and inhibits the activation of GSK-3β, which promotes the transcriptional activation of C-myc^16^,^17^,^18^,^19^. The mammalian NF-κB family includes p50 (NF-κB1), p52 (NF-κB2), p65 (ReLA, NF-κB3), ReL and ReLB, which share the amino-terminal ReL homology domain RHD and are regulated by the eight IκB family members^20^. Previous studies have shown that AEG-1 is an important positive regulator of nuclear factor kappa-B p65 (NF-κB) and that the activation of NF-κB p65, which is induced by AEG-1, exhibits a key molecular mechanism in which AEG-1 promotes cell growth and invasion in malignant glioma cells^8^,^21^.

DYT-40 (referred to as compound 3c in a previous study) is a novel 2-styryl-5-nitroimidazole derivative containing the 1,4-benzodioxan moiety (3a-3r). These compounds (3a-3r) have been synthesized, biologically evaluated, and demonstrated to be FAK inhibitors in molecular docking studies^22^. Among all compounds, 3p exhibits significant FAK inhibitory activity (IC_50_ = 0.45 μM) and possesses good A549 anti-proliferative activity. However, the FAK inhibitory effect of compound 3c (DYT-40, IC_50_ = 18.42 μM) is not as good as that of compound 3p. Although 3p showed the most potent activity *in vitro* which inhibited the growth of adenocarcinomic human alveolar basal epithelial cells A549 with IC_50_ value of 3.11 μM and human cervical cancer cells Hela with IC_50_ value of 2.54 μM respectively, the efficacy of DYT-40 on glioma cells growth seems to be better than 3p.

The present study provides the first evidence that DYT-40 represses the expression of AEG-1 and the activation of the NF-κB pathway, which plays an important role in tumor development and progression^6^,^8^.

## Materials and Methods

### Cell lines and culture conditions

Human malignant glioma U251 and U87 cells were obtained from Cell Bank of Shanghai Institute of Biochemistry and Cell Biology, Chinese Academy of Sciences, and cultured in DMEM (Gibco, Grand Island, NY, USA) medium containing 10% fetal bovine serum (Gibco, Grand Island, NY, USA)^23^. The cells were grown at 37 °C in a humidified atmosphere of 5% CO_2_.

### Reagents and antibodies

Primary antibodies against AEG-1/MTDH (13860-1-AP), E-cadherin (1702-1), N-cadherin (2447-1), and vimentin (2862-1) were obtained from Epitomics Inc. (Burlingame, CA, USA). Primary antibodies against poly-ADP-ribose polymerase (PARP) (BS70001), PI3K (BS3006), Bax (BS1030), Bcl-2 (BS1031), and C-myc (BS2462) were obtained from Bioworld Technology Inc. (Minneapolis, MN, USA). Primary antibodies against NF-κB-p65 (10745-1-AP), caspase-8 (13423-1-AP), caspase-9 (10380-1-AP), and caspase-3 (19677-1-AP) were obtained from Proteintech (Wuhan, China); Primary antibodies against Lamin A (sc-177452), AKT (sc-8312), p-AKT (sc-7985), ERK (sc-154), and p-ERK (sc-23759) were obtained from Santa Cruz Biotechnology (Santa Cruz Biotechnology Inc., CA). The GAPDH antibody was purchased from Boster (Wuhan, China). The secondary antibodies peroxidase-conjugated AffiniPure Goat Anti-Mouse IgG (H+L) (111-035-003), peroxidase-conjugated AffiniPure Goat Anti-Rabbit IgG (H+L) (111-035-003), Cy™ 3-conjugated AffiniPure Goat Anti-Rabbit IgG(H+L) (111-165-003), and Fluorescein (FITC)-conjugated AffiniPure Goat Anti-Rabbit IgG(H+L) (111-095-003) were obtained from Jackson Immuno Research Laboratories, Inc. (USA). Temozolomide (85622-93-1) was obtained from Meilun Biology Technology (Dalian, China).

### Synthesis of compound DYT-40

**Figure 1:**
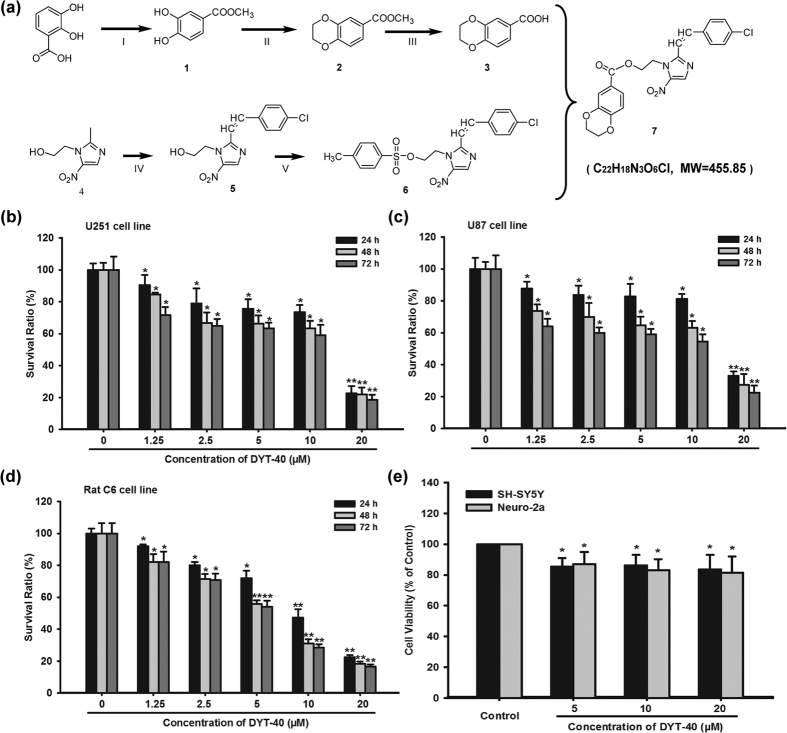
The synthetic routes and cell viability inhibition effect of DYT-40. (a) The synthetic routes of DYT-40, (E)-2-(2-(4-chlorostyryl)-5-nitro-1H-imidazol-1-yl) ethyl-2-(2, 3-dihydrobenzo [b] [1,4] dioxin-6-yl) acetate (C_22_H_18_N_3_O_6_Cl, MW = 455.85). **(b**–**d)** Cell viability was determined by Cell Counting Kit-8 (CCK-8) assay. U251, U87 and C6 cells were treated with 0, 1.25, 2.5, 5, 10 and 20 μM DYT-40 for 24 h, 48 h and 72 h, respectively. CCK-8 assay was used to measure cell viability. **(e)** The human SH-SY5Y cells and mouse Nuro2a cells were treated with 0, 5, 10 and 20 μM DYT-40 for 48 h, respectively. CCK-8 assay was used to measure cell viability.

The synthesis of DYT-40, a novel synthetic (E)-2-(2-(4-chlorostyryl)-5-nitro-1H-imidazol-1-yl)ethyl-2-(2,3-dihydrobenzo[b][1,4]dioxin-6-yl)acetate targeting AEG-1 (PDB: 4QMG), is outlined in Fig. 1a^22^. The compound 2,3-dihydrobenzo[1,4]dioxine-6-carboxylic acid was prepared in three steps. First, methyl 3,4-dihydroxybenzoate was generated from 3,4-dihydroxybenzoic acid using concentrated sulfuric acid in methanol. Second, the treatment of compound 1 with dibromoethane in acetone produced compound 2, which was subsequently saponified (NaOH (*aq*), MeOH, and THF), yielding 2,3-dihydrobenzo[1,4]dioxine-6-carboxylic acid (compound 3). Compound 5 was synthesized by the reaction of metronidazole with 4-Cl-benzaldehyde in DMSO via the rapid addition of a stirred solution of sodium methoxide in methanol at room temperature. Compound 5 was treated with 4-methylbenzoyl chloride using triethylamine as catalyst in CH_2_Cl_2_, thus generating compound 6. Compound 6 was refined through subsequent purification with chromatography. Subsequently, 2,3-dihydrobenzo[1,4]dioxine-6-carboxylic acid, compound 6, and K_2_CO_3_ were combined in DMF and refluxed to provide compound 7, named DYT-40 (Fig. 1a). DYT-40 (purity > 98%) was prepared as a 10^5^ μmol/L (μM) stock solution in sterilized dimethylsulfoxide (Sigma, St. Louis, MO, USA) and stored at −20 °C.

### Cell proliferation assay

Cell growth was analyzed using Cell Counting Kit-8 (Dojindo Molecular Technologies, Inc., Japan) according to the manufacturer’s instructions^24^. Briefly, the cells were seeded at a density of 5 × 10^3^ cells/well onto 96-well plates and treated with various concentrations of DYT-40 (0.005, 0.05, 0.5, 5 and 50 μM) and cultured for 24, 48 and 72 h at 37 °C in 5% CO_2_. Subsequently, the medium in each well was substituted with 100 μL of fresh medium containing 10% Cell Counting Kit-8, and the cultures were incubated at 37 °C for 2 h. The absorbance value (A) was determined using a Synergy™ 2 Multi-Mode Microplate Reader (BioTek Instruments, Inc., Winooski, VT, USA) at 450 nm. The Survival Ratio (%) = average absorbance of treated group/average absorbance of control group ×100. The IC_50_ was defined as the concentration of DYT-40 that reduced cell viability by 50%, calculated using the logit method. All assays were performed in triplicate.

### Annexin V/PI Staining

The Annexin V/PI Staining Apoptosis-mediated cell death of U251 and U87 cells was examined using the Annexin V-FITC Apoptosis Detection Kit I (Catalog Number: 556547, BD Biosciences, Qume Drive, San Jose, CA, USA) according to the manufacturer’s instructions. Briefly, 1 × 10^6^ cells were harvested and washed with PBS. Subsequently, the cells were re-suspended in 500 μL of binding buffer, and 5 μL Annexin-V-FITC and 1 μL PI were added, followed by incubation for 10 min in the dark at room temperature^23^,^25^. The cells were analyzed using the BD FACSCalibur™ Flow Cytometry System (BD Biosciences, Franklin Lakes, NJ) and the Cell Quest software (BD Biosciences). Cells in the early stages of apoptosis were Annexin V positive and PI negative, whereas cells in the late stages of apoptosis were both Annexin V and PI positive.

### DAPI staining assay

To observe the morphology of apoptosis, the cell nuclei were visualized following DNA staining. Briefly, the cells were seeded onto six-well tissue culture plates at a concentration of 1 × 10^5^ cells/well and treated with the indicated concentrations of DYT-40 (0, 5, 10 and 20 μM). In addition, cells pretreated or not with pcDNA3.1-AEG-1 (1 μg) were incubated with 20 μM DYT-40 and subsequently fixed with 4% paraformaldehyde for 20 min and washed with PBS, followed by incubation with the fluorochrome dye 4′,6-diamidino-2-phenylindole (DAPI, 1 μg/mL, Sigma-Aldrich) for 10 min. After washing with PBS, the cells were observed using fluorescence microscopy (Olympus, Tokyo, Japan) with a peak excitation wavelength of 340 nm^25^.

### Cell adhesion assay

The cell adhesion assay was performed as previously described^26^, with some modifications. The 96-well plates were coated with fibronectin (5 μg/mL) at 4 °C overnight and subsequently blocked in BSA (1%) for 1 h. Serum-starved cells were exposed to DYT-40 (2.5, 5, or 10 μM) for 24 h prior to seeding. The target cells were harvested and suspended in serum-free medium. The cells (2 × 10^5^/mL) were seeded onto fibronectin-coated plates and subsequently incubated at 37 °C for 1 h. Non-adherent cells were removed after gentle washing with PBS. Subsequently, the colorimetric MTT assay was employed to analyze the cell adhesion.

### Transwell invasion assay

The transwell migration assay was performed using 24-well MILLI Cell Hanging Cell Culture inserts 8 mm PET (Millipore, Bedford, MA, USA) as previously described^27^. The cells were cultured in DYT-40 (2.5, 5 and 10 μM) for 24 h, and in parallel, pcDNA3.1-AEG-1 was transfected into U251 and U87 cells for 24 h and treated with DYT-40 (10 μM) for an additional 24 h. Subsequently, the cells were seeded in serum-free medium onto triplicate wells of BD BioCoat^TM^ Matrigel^TM^ Invasion Chambers (BD Bioscience), and complete medium containing 10% fetal bovine serum was added to the lower chamber. The cells invaded through the polycarbonate membrane were stained using crystal violet after incubation for 24 h. Image magnification: 200×. Five random fields from each of the triplicate invasion assays were counted using phase-contrast microscopy.

### Transient transfection

The pcDNA3.1-AEG-1/MTDH plasmid was kindly provided from Dr. Kunmei Liu (Ningxia Medical University)^28^. For transfection, the cells were trypsinized and seeded onto 6-well plates at a density of 5000 cells/well. The plasmids were transfected into the cells at 1 μg/well using Lipofectamine^TM^ 2000 transfection reagent (Invitrogen, Carlsbad, CA) according to the manufacturer’s instructions^25^. The expression vector was transfected at 24 h prior to reseeding onto 96-well plates. The AEG-1/MTDH siRNA oligonucleotides (Sequence 1: sense 5′-GCAGCAAGGCAGTCTTTAAGT-3′, antisense 5′-ACTTAAAGACTGCCTTGCTGC-3′; Sequence 2: sense 5′-GUUACCACCGAGCAACUUAdTdT-3′, antisense 5′-UAAGUUGCUCGGUGGUAACdTdT-3′) were synthesized at GenePharma, Inc. (Pudong, Shanghai). A universal siRNA was used as a negative control. We performed siRNA transfection using Lipofectamine^TM^ 2000 (Invitrogen, Carlsbad, CA) according to the manufacturer’s instructions. Briefly, cells at 50% confluence were transfected with serum-free DMEM medium containing 100 nM scrambled siRNA, AEG-1 for 6 h, followed by recovery in medium containing serum and other special treatments as needed.

### Quantitative real-time polymerase chain reaction

Total RNA was isolated using TRIzol reagent, according to the manufacturer’s instructions. First-strand cDNA was synthesized using total RNA (1 μg) at 70 °C for 5 min, 42 °C for 60 min, and 95 °C for 10 min using an oligo (dT) 12–18 primer and subjected to real-time PCR. Amplification assays were performed on a 7900 Fast Real-Time PCR System using the SDS software (Applied Biosystems) in 10-μL reactions containing 0.2 μM of each primer, 5 μL SYBR Green PCR master mix (2×) and 0.2 μL cDNA. After an initial denaturation at 95 °C for 30 s, amplifications were performed for 40 cycles at 95 °C for 5 s and 60 °C for 31 s. The signals from each target gene were normalized based on the corresponding GAPDH signal. The following PCR primers were used in the present study: (1) AEG-1 F: CTAGTATCCTGGTTTAACAACAGTGCCCTGTTTACAACAGATTGTGCCCTATCTCATCA, AEG-1 R: AGCTTGATGAGATAGGGCACAATCTGTTGTAAACAGGGCACTGTTGTTAAACCAGGATA; (2) GAPDH F: CAGTCCATGCCATCACTGCCA, GAPDH R: CAGTGTAGCCCAGGATGCCCTT.

### Western blotting analysis

Briefly, after washing twice with PBS, the cultured cells were collected and lysed in lysis buffer (100 mM Tris-HCl, pH 6.8, 4% (m/v) SDS, 20% (v/v) glycerol, 200 mM β-mercaptoethanol, 1 mM phenylmethylsulfonyl fluoride, and 1 g/mL aprotinin). Nuclear proteins were extracted using a nuclear and cytoplasmic protein extraction kit (KeyGen, Nanjing, China) according to the manufacturer’s instructions. The lysates were centrifuged at 13,000 × *g* for 15 min at 4 °C. The concentration of total proteins was measured using the BCA assay method with a Varioskan spectrofluorometer and spectrophotometer (Thermo, Waltham, MA) at 562 nm^25^. The protein samples were separated on a 12% SDS-PAGE gel and electrophoretically transferred onto polyvinylidene difluoride (PVDF) membranes (Millipore, Boston, MA). The immune complexes were formed after the incubation of the proteins with primary antibodies overnight at 4 °C. After incubation with the appropriate secondary antibodies, the blots were visualized using ECL plus western blotting detection reagents (Bio-Rad) and a ChemiDoc XRS Plus luminescent image analyzer (Bio-Rad, Hercules, CA, USA). The densitometric analysis of the band intensity was performed using Image lab software (Bio-Rad, Hercules, CA, USA).

### Immunofluorescence and confocal fluorescence microscopy

Cells pretreated with pcDNA3.1 (1 μg) and pcDNA3.1-AEG-1 (1 μg) were incubated with 10 μM DYT-40 and vehicle, respectively, for 24 h. Subsequently, the cells were fixed with 4% paraformaldehyde in PBS at 1-hour intervals, permeabilized with 0.5% Triton X-100, and blocked with 2% BSA for 30 min, followed by incubation with primary antibodies (diluted 1:50) against AEG-1 and NF-κB overnight at 4 °C. Subsequently the nuclei were stained with 4′,6-diamidino-2-phenylindole (DAPI, Sigma-Aldrich) for 10 min prior to imaging. A FV10-ASW laser scanning confocal microscope [Ver 2.1] (Olympus Corp, MPE FV1000) was used for co-localization analysis^23^.

### Animals

Adult male Sprague-Dawley rats (Charles River Laboratories, Sulzfeld, Germany) weighing 200–250 g were raised in a specific pathogen free (SPF) grade animal laboratory. The animals were housed in groups of two under standard conditions at a temperature of 22 °C ± 1 and a 12 h-12 h light/dark cycle starting at 7:00 AM with free access to food and water. All experiments were conducted according to the National Institutes of Health Guide for the Care and Use of Laboratory Animals (publication no. 85–23, revised 1985) and approved by the IACUC (Institutional Animal Care and Use Committee of Nanjing Medical University, Ethical NO.14030134).

### Orthotopic glioma model

Prior to implantation, 85–90% confluent C6 cells were trypsinized, rinsed with F12K+10% fetal calf serum, and centrifuged at 1000 rpm for 4 min. The cell pellet was resuspended in F12K and placed on ice. The concentration of viable cells was adjusted to 1 × 10^8^ cells/mL in F12K. Each rat was anesthetized by a peritoneal injection with 0.4 mL/100 g of a 1% pentobarbital sodium solution and placed in a stereotactic frame (RWD Life Science, Shenzhen, China). After shaving and disinfection of the skin, the skull was exposed using a sagittal incision, and a burr hole 1 mm anterior and 3 mm lateral relative to the bregma was produced using a small drill. The cell suspension was injected into a depth of 6 mm from the skull surface, using a 2-μL Hamilton (#2701) syringe (Reno, NV, USA) with a 26s-gauge needle mounted on a stereotactic holder^29^. After injection, the needle was left in place for 5 min and slowly withdrawn. The scalp incision was subsequently closed with surgical sutures. The animals were intramuscularly injected with 0.1 mL/rat of 80 U/mL benzylpenicillin sodium solution to prevent infection and returned to their home cages. The rats were divided into five groups. The negative control group was treated with vehicle. The positive control was intravenous (i.v.) injected with 10 mg/kg temozolomide. The other three groups were intravenously injected with DYT-40 (5, 10, 20 mg/kg) at 24 h after surgery, respectively. All drugs were administered once a day and continued for two weeks. The maximum cross-sectional area of the intracranial glioblastoma xenografts was determined by computer-assisted image analysis using a Leica Quantimet 500-system (Leica, Hamburg, Germany). The tumor volume was calculated as the square root of the maximal tumor cross-sectional area^3^,^30^.

### Histology, immunohistochemistry assay

The animals were deeply anesthetized and transcardially perfused with 4% paraformaldehyde in phosphate-buffered saline (PBS). The brains were removed from the skulls and post-fixed overnight at 4 °C in 4% paraformaldehyde. On the next day, the brains were transferred to 30% sucrose in PBS solution for 48 h at 4 °C. Coronal sections with a thickness of 10 mm were cut using a cryostat microtome (Leica CM1900, Germany). Hematoxylin and eosin (H&E) staining was used to visualize the tumor area and tumor necrosis. To evaluate cell proliferation, immunostaining for AEG-1 was used. The antibody against AEG-1 was diluted 1:300 in blocking solution containing 0.3% Triton X-100 and incubated overnight at 4 °C. After washing in PBS, the sections were incubated with a biotinylated secondary antibody for 2 h, followed by washing and further incubation with a streptavidin-biotin-peroxidase complex (Vector Laboratories). Apoptosis was examined using the terminal deoxynucleotidyl transferase-mediated deoxyuridine triphosphate nick-end labeling (TUNEL) method (*In Situ* Cell Death Detection Kit; Roche Molecular Biochemicals) according to the manufacturer’s instructions.

### Statistical analysis

All statistical analyses were performed using SPSS 10.0 for Windows software package (SPSS Inc., Chicago, IL, USA). The data are represented as the mean ± SEM of three independent experiments. Statistical comparisons of the results were performed using analysis of variance. *^,#^p < 0.05 compared with control; **^,##^p < 0.01 compared with control. ^$$,&&^p < 0.01 compared with DYT-40 (10 μM)-treated cells.

## Results

### DYT-40 inhibited the proliferation of malignant glioblastoma cells

The ability of DYT-40 to inhibit the growth of human malignant glioma cells was investigated using the CCK-8 assay. As shown in Fig. 1b, c and d, DYT-40 inhibited cell growth in U251, U87 and C6 cells in a concentration- and time-dependent manner. The inhibitory effects were apparent at concentrations of 1.25, 2.5, 5, 10 and 20 μM of DYT-40, and cytocidal effects were observed under these conditions. The IC_50_ values of DYT-40 in U251 cells were 11.78, 8.55 and 6.52 μM for 24, 48 and 72 h of treatment, respectively. The IC_50_ values of DYT-40 in U87 cells were 22.05, 9.65 and 5.53 μM for 24, 48 and 72 h of treatment, respectively. The IC_50_ values of DYT-40 in C6 cells were 8.55, 5.34 and 5.02 μM for 24, 48 and 72 h treatment, respectively. However, as shown in Fig. 1e, DYT-40 at concentration of 5, 10, 20 μM showed little effect on the viability of human neuroblastoma SH-SY5Y cells (85.35 ± 5.57%, 86.24 ± 6.93%, 83.48 ± 9.65%) and mouse neuroblastoma Neuro2a cells (87.01 ± 7.92%, 83.02 ± 7.07%, 81.44 ± 10.47%).

DYT-40 at concentration of 1.25, 2.5, 5, 10, 20 μM showed little effect on the viability of MCF10A (human normal mammary epithelial cells) and HEK293T (human embryonic kidney 293T cells) As shown in Fig. S1, DYT-40 at concentration of 1.25, 2.5, 5, 10, 20 μM showed little effect on the viability of MCF10A cells (93.21 ± 11.16%, 89.41 ± 8.00%, 87.38 ± 7.66%, 83.76 ± 8.74%, 79.21 ± 7.64%) and HEK293T cells (99.41 ± 0.84%, 94.33 ± 2.39%, 91.63 ± 2.26%, 87.94 ± 3.27%, 84.14 ± 4.47%). We have discussed the supplemental data in revised the text. As shown in Fig. S2, temozolomide inhibited cell growth in C6 cells in a concentration- and time-dependent manner. The inhibitory effects were apparent at concentrations of 1.25, 2.5, 5, 10 and 20 μM of temozolomide. The IC_50_ values of temozolomide in C6 cells were 12.90, 11.16 and 6.82 μM for 24, 48 and 72 h treatment, respectively. Thus, the efficacy of DYT-40 on glioma C6 cells growth seems to be better compared to temozolomide.

As shown in Fig. S3a, 3p at concentration of 1.25, 2.5, 5, 10, 20 μM showed inhibitory effect on the viability of U251 cells (85.03 ± 7.44%, 80.04 ± 5.66%, 75.86 ± 3.42%, 65.14 ± 2.62%, 51.25 ± 8.88%) and U87 cells (94.47 ± 6.68%, 87.93 ± 1.70%, 80.38 ± 5.80%, 67.18 ± 5.97%, 57.51 ± 7.75%). The IC_50_ values of 3p in U251 and U87 cells were more than 20 μM for 72 h treatment. As shown in Fig. S3b, after treated with 10 and 20 μM 3p for 24 h, the early and median apoptotic cells (right lower section of fluorocytogram) were determined to be 3.74% and 4.01% (U251), 8.48% and 8.61% (U87), whereas, the control was only 1.33% (U251) and 6.44% (U87), respectively. Meanwhile, the late apoptotic and necrotic cells (right upper section of fluorocytogram) were determined to be 10.76% and 11.94% (U251), 4.32% and 6.23% (U87), whereas the control was only 1.16% (U251) and 1.14% (U87), respectively. Thus, the efficacy of DYT-40 on glioma cells growth seems to be better than 3p.

### DYT-40-mediated inhibition of AEG-1 expression contributed to the growth-inhibitory effect of malignant glioblastoma cells

**Figure 2:**
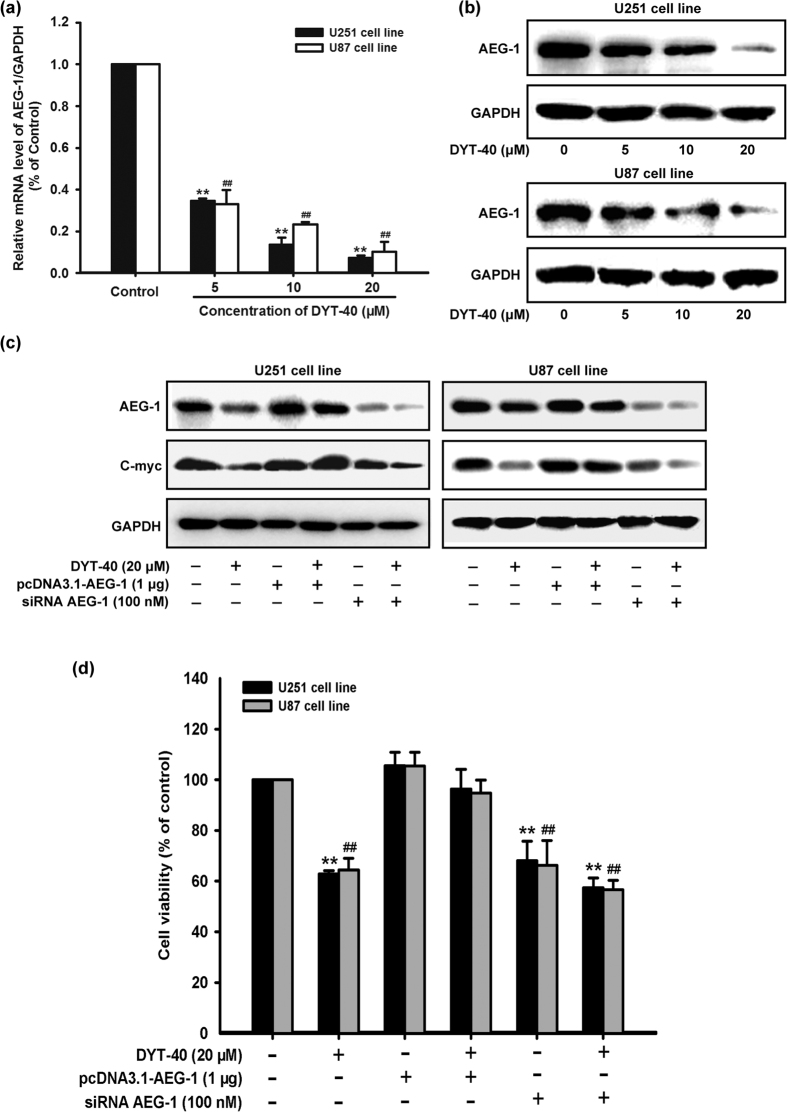
Inhibition of AEG-1 level by DYT-40 participated in growth suppression in U251 and U87 cells. (**a**) U251 and U87 cells were treated with the indicated concentrations of DYT-40 for 24 h. AEG-1 mRNA levels were evaluated by Q-real time PCR and normalized to GAPDH. **(b)** U251 and U87 cells were treated with the indicated concentrations of DYT-40 for 24 h. AEG-1 protein expression were evaluated by Western blotting. **(c)** pcDNA3.1 (1 μg) and pcDNA3.1-AEG-1 (1 μg) were transfected into U251 and U87 cells for 48 h. Si control (100 nM) and siRNA AEG-1 (100 nM) were transfected into U251 and U87 cells for 48 h. Cells were harvested and cell lysates were extracted to analyze the expression of AEG-1, C-myc and GAPDH by Western blotting. **(d)** U251 and U87 cells were transfected with pcDNA3.1-AEG-1 (1 μg) and siRNA AEG-1 (100 nM) for 24 h and reseeded to 96-well plates as mentioned, then cells were treated with DYT-40 (20 μM) and subjected for a 3-day CCK-8 assay. Data are presented as the mean ± SEM of three separate expriments. *^,#^p < 0.05, and **^,##^p < 0.01, represent a statistically significant decrease in response to Control group.

To further investigate the inhibitory effect of DYT-40 on the malignant glioma cell growth, we examined changes in AEG-1 expression after DYT-40 (5, 10 and 20 μM) treatment for 24 h. As shown in Fig. 2a, after U251 and U87 cells were treated with different concentrations of DYT-40 for 24 h, the AEG-1 mRNA levels were monitored by Q-real time PCR. The AEG-1 mRNA levels were decreased in a concentration-dependent manner in response to DYT-40. Western blotting was used to monitor the expression of AEG-1 protein after DYT-40 treatment. The AEG-1 protein levels decreased after DYT-40 treatment in a concentration-dependent manner (Fig. 2b).

To elucidate whether AEG-1 expression is involved in the inhibition of DYT-40 cell growth, U251 and U87 cells were transfected with AEG-1 siRNA and pcDNA3.1-AEG-1, followed by DYT-40 (20 μM) treatment for an additional 24 h. The Knockdown of AEG-1 expression further reduced DYT-40-mediated decrease in AEG-1 and C-myc expression (Fig. 2c). Conversely, the over-expression of AEG-1 rescued DYT-40-induced AEG-1 and C-myc expression (Fig. 2c). As shown in Fig. 2d, the cell viability with DYT-40 treatment was 62.77 ± 1.39% (U251) and 64.34 ± 4.72% (U87). The cell viability with DYT-40 treatment was rescued 96.35 ± 7.76% (U251) and94.76 ± 5.06% (U87) via the over-expression of AEG-1. The cell viability was inhibited 57.42 ± 3.89% (U251) and 56.61 ± 3.70% (U87) after DYT-40 treatment combined with siRNA AEG-1.

### DYT-40 induced apoptosis in malignant glioblastoma cells

**Figure 3:**
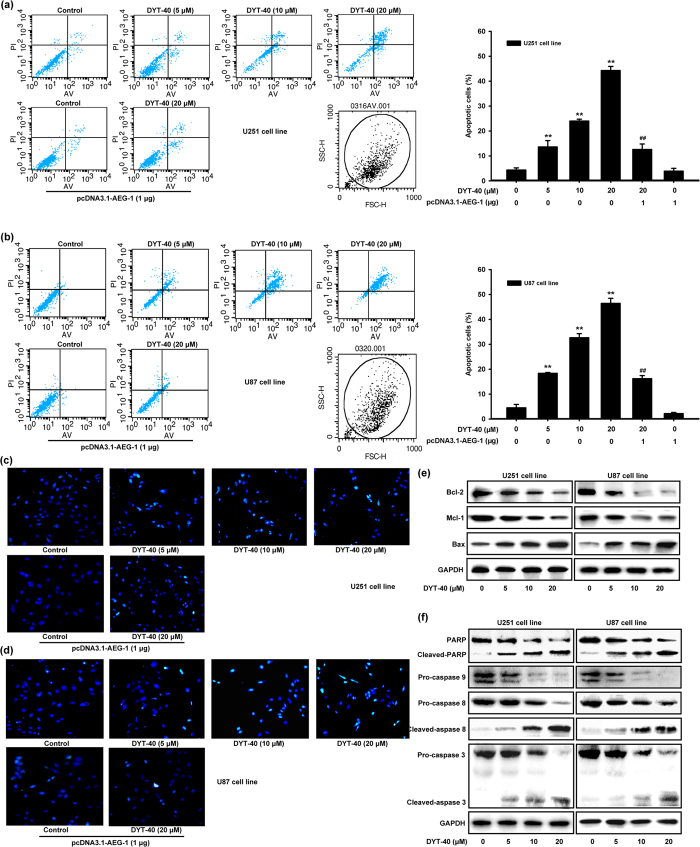
DYT-40 induced apoptosis in U251 and U87 cells. **(a**,**b)** Effect of treatment of DYT-40 (5, 10, and 20 μM) for 24 h on the apoptosis of cells was determined. On the other hand, pcDNA3.1-AEG-1 were transfected into U251 and U87 cells for 24 h, and cells were treated with DYT-40 (20 μM) for an additional 24 h. The sum of early and late apoptotic cells ratio (%) were quantitated by flow cytometer analysis of Annexin V/PI. Results from three independent experiments are shown as means ± SEM. **P < 0.01, ^##^P < 0.01, control group cells versus combination of DYT-40 and pcDNA3.1-AEG-1-treated cells. **(c**,**d)** DAPI staining assay was used to detect the apoptosis, after U251 and U87 cells were treated with DYT-40 (5, 10, and 20 μM) for 24 h. In parallel, pcDNA3.1-AEG-1 were transfected into U251 and U87 cells for 24 h, and cells were treated with DYT-40 (20 μM) for an additional 24 h. DAPI staining assay was also used to detect the apoptosis. **(e**,**f)** the expression of apoptosis related protein Bax, Bcl-2, Mcl-1, PARP, caspase-3, caspase-8, and caspase-9 was assessed by Western blotting in U251 and U87 cells treated with DYT-40 (5, 10, and 20 μM) for 24 h.

The effect of DYT-40 (5, 10 and 20 μM) treatment for 24 h on the apoptosis of cells was determined. To this end, pcDNA3.1-AEG-1 was transfected into U251 and U87 cells for 24 h, and the cells were treated with DYT-40 (20 μM) for an additional 24 h. Subsequently, DYT-40-induced apoptosis was analyzed by Annexin V/PI staining. As shown in Fig. 3a,b, after treatment with 5, 10 and 20 μM DYT-40 for 24 h, the early and median apoptotic cells (right lower section of fluorocytogram) were determined as 8.62%, 11.49% and 20.96% (U251) and 8.92%, 17.47% and 21.80% (U87), respectively, whereas the control was only 1.52% (U251) and 1.29% (U87). In addition, the late apoptotic and necrotic cells (right upper section of fluorocytogram) were determined to be 4.98%, 12.54% and 23.32% (U251) and 9.41%, 15.16% and 24.65% (U87), respectively, whereas the control was only 2.8% (U251) and 3.2% (U87). Moreover, pretreatment with pcDNA3.1-AEG-1 (1 μg) rescued early and median apoptotic cells with DYT-40 (20 μM) treatment from 20.96% to 7.8% (U251) and 21.80% to 7.26% (U87) and late apoptotic and necrotic cells were rescued from 23.32% to 4.72% (U251) and 24.65% to 8.96% (U87). After treatment with DYT-40 (5, 10 and 20 μM) for 24 h or treatment with DYT-40 (20 μM) for an additional 24 h followed by pcDNA3.1-AEG-1 transfection into U251 and U87 cells for 24 h, the cells presented morphological features of early apoptosis, including bright, nuclear condensation in the DAPI staining assay (Fig. 3c,d). These features appeared more frequent with increasing concentrations of DYT-40 and were rescued after pcDNA3.1-AEG-1 transfection.

We evaluated the expression of the apoptotic protein Bax, the anti-apoptotic proteins Bcl-2 and Mcl-1, caspases 3, 9 and 8, and PARP using western blotting. The results showed that Bax expression increased while expression Bcl-2 decreased. Thus, the ratio of Bax/Bcl-2, which is crucial for the activation of the mitochondrial apoptotic pathway, increased in cells treated with DYT-40. In addition, Mcl-1, an anti-apoptotic Bcl-2 homolog, decreased after DYT-40 treatment (Fig. 3e). As shown in Fig. 3f, PARP cleavage increased as the concentration of DYT-40 increased. We further examined the involvement of caspases in DYT-40-mediated apoptosis. Compared with the controls, caspase-3, caspase-9 and caspase-8 were all activated after DYT-40 treatment for 24 h. These results demonstrated that the intrinsic apoptotic pathway is involved in DYT-40-induced apoptosis in U251 and U87 cells and that antitumor activity might be ascribed to apoptosis induction.

### DYT-40 suppressed the invasion and epithelial-mesenchymal transition of malignant glioblastoma cells

**Figure 4:**
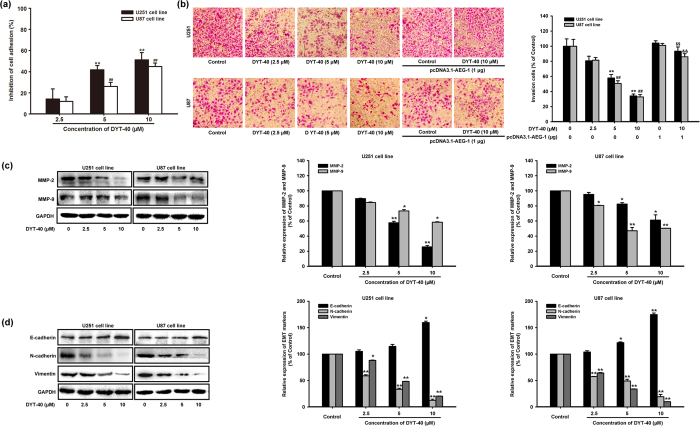
DYT-40 inhibited epithelial-mesenchymal transition and invasion of U251 and U87 cells. (**a**) Inhibitory effect of DYT-40 (2.5, 5, and 10 μM) on adhesion of U251 and U87 cells to fibronectin. Cell suspension (100 μL, 2 × 10^5^ cells/mL) was added to fibronectin pre-coated plates and incubated at 37 °C for 1 h. Then culture media was carefully suctioned out. Each well was washed three times with PBS. MTT assay was adopted to determine the number of adherent cells. **(b)** DYT-40 inhibits U251 and U87 cell invasion. Cells were cultured in DYT-40 (2.5, 5, and 10 μM) for 24 h, in parallel, pcDNA3.1-AEG-1 were transfected into U251 and U87 cells for 24 h, and cells were treated with DYT-40 (10 μM) for an additional 24 h. Then cells were seeded in the upside of transwell coated with matrigel, the cells invaded through the polycarbonate membrane were stained by crystal violet after 24 h of incubation. Image magnification: 200×. **(c)** cells were treated with DYT-40 (2.5, 5, and 10 μM) for 24 h, and the protein levels of MMP-2 and MMP-9 in U251 and U87 cells were detected by Western blotting using specific antibodies and GAPDH was used as loading control. **(d)** Effect of DYT-40 on the protein expression of epithelial-mesenchymal transition markers in U251 and U87 cells. Western blotting was performed with antibodies specific for E-cadherin, vimentin, and N-cadherin after cells were treated with DYT-40 (2.5, 5, and 10 μM) for 24 h. Each bar represents the mean ± SEM calculated from three independent experiments. *^,#^p < 0.05 compared with control; **^,##^p < 0.01 compared with control. ^$$,&&^p < 0.01 compared with DYT-40 (10 μM)-treated cells.

Tumor cell adhesion to the ECM and basement membrane was considered as a fundamental step for cancer metastasis. We examined the influence of DYT-40 on the adhesion of U251 and U87 cells to substrates that were precoated with fibronectin, which is an important component of the ECM. DYT-40 treatment (2.5, 5 and 10 μM) suppressed U251 and U87 cell adhesion to fibronectin by 14.22 ± 9.57%, 41.76 ± 3.99 and 51.16 ± 6.87% and by 11.92 ± 4.32%, 25.93 ± 4.01 and 44.89 ± 3.41%, respectively (Fig. 4a). Moreover, the effects of DYT-40 (2.5, 5 and 10 μM) treatment for 24 h on the invasion of cells were determined. pcDNA3.1-AEG-1 was transfected into U251 and U87 cells for 24 h, and the cells were treated with DYT-40 (10 μM) for an additional 24 h. Subsequently, the cells were seeded onto the upside of transwell coated with matrigel, and the cells that invaded through the polycarbonate membrane were stained using crystal violet after 24 h of incubation. As shown in Fig. 4b, DYT-40 treatment reduced the invasion of U251 and U87 cells in a concentration-dependent manner. Cell invasion was rescued by pcDNA3.1-AEG-1 transfection.

Previous studies have shown that MMP-2 and MMP-9 play a critical role in cancer cell invasion by stimulating the degradation of the ECM^31^. The protein levels of MMP-2 and MMP-9 in DYT-40-treated U251 and U87 cells were decreased 25.75 and 58.54% (U251), 61.32 and 50.40% (U87), respectively, suggesting that DYT-40 suppresses the invasion ability of U251 and U87 cells by down-regulating the activity and expression of MMP-2/9 (Fig. 4c). To further investigate the effect of DYT-40 on malignant cell invasion, we examined the changes in epithelial and mesenchymal markers in DYT-40 treatments from 2.5 to 10 μM at 24 h. As shown in Fig. 4d, DYT-40 increased the E-cadherin protein levels, whereas the N-cadherin and vimentin protein levels were decreased in U251 and U87 cells. Compared with the control groups, 10 μM DYT-40 increased the E-cadherin levels by 159.69 and 174.68%. In contrast, the N-cadherin and vimentin protein levels were reduced 12.59 and 20.56% (U251) and 19.53 and 9.85% (U87), respectively.

### DYT-40 suppressed AEG-1/NF-κB, PI3K/AKT and MAPK pathways

**Figure 5:**
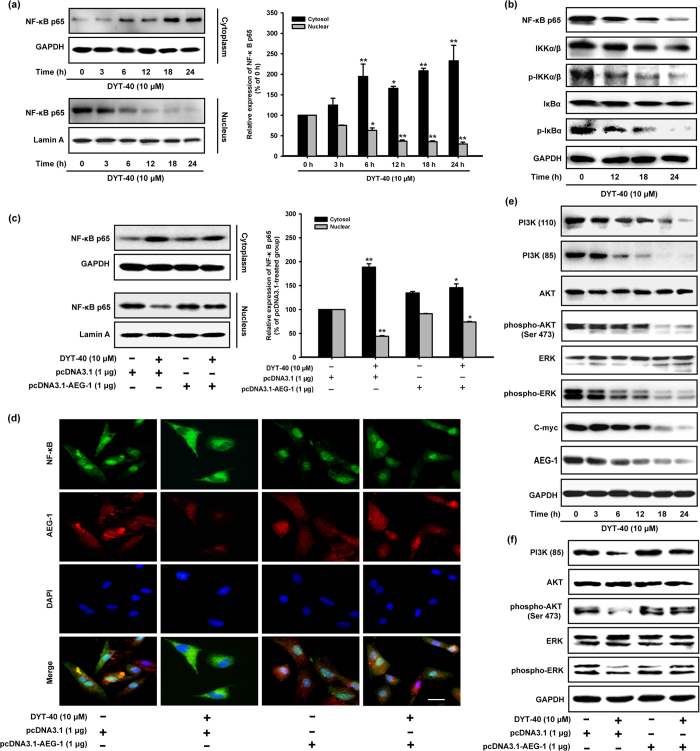
DYT-40 suppressed AEG-1/NF-κB, PI3K/AKT and MAPK pathways. **(a)** Cytoplasm fractions and nucleus extracts were prepared from the cells treated with DYT-40 (10 μM) for the indicated times (0, 3, 6, 12, 18, and 24 h). The expression of NF-κB p65 was determined as shown by Western blotting. *p < 0.05 and **p < 0.01. (**b**) Cells were treated with DYT-40 (10 μM) for the indicated times (0, 12, 18, and 24 h). The phosphorylation levels of IKKα/β and IκBα was detected by Western blotting using specific antibodies where GAPDH was used as loading control. **(c)** The pcDNA3.1 (1μg) and pcDNA3.1-AEG-1 (1 μg) were transfected into U87 cells for 24 h, and cells were treated with DYT-40 (10 μM) for an additional 24 h. After the isolation of nucleus and cytoplasm extracts, the levels of protein were measured by Western blotting. *p < 0.05 and **p < 0.01. **(d)** Nuclear translocation of NF-κB p65 and expression of AEG-1 were determined by triple-label immunofluorescence confocal microscopy. The cells were fixed and stained with rabbit anti-AEG-1, followed by Alexa Fluor 568-conjugated anti-rabbit antibody (red); NF-κB p65 protein expression was stained with rabbit anti-NF-κB p65 antibody (green) and secondary antibody goat anti-rabbit IgG/FITC. The nuclei were visualized by staining with DAPI (blue). The merged images represent the areas within the yellow squares and highlight the relative location of NF-κB p65 to AEG-1. **(e)** PI3K/AKT and MAPK signaling pathways accompanied with C-myc/AEG-1 were detected, and GAPDH was used as loading control. **(f)** The pcDNA3.1 (1 μg) and pcDNA3.1-AEG-1 (1 μg) were transfected into U87 cells for 24 h, and cells were treated with DYT-40 (10 μM) for an additional 24 h. The PI3K/AKT and MAPK signaling pathways were measured by Western blotting.

Nuclear factor kappa B (NF-κB) exerted an anti-apoptotic effect via the induction of genes that inhibit apoptotic signaling pathways^32^. In addition, AEG-1 induced the invasiveness of tumor cells and increased the expression of adhesion molecules through the activation of the NF-κB signaling pathway^8^. AEG-1 also physically interacted with p65 to modulate the function of this protein in the nucleus^12^. The activation and translocation of p65, which is the functional active subunit of NF-κB, were analyzed when U87 cells were treated with DYT-40. The nuclei were isolated, and the amount of NF-κB p65 in the nuclear fraction was quantified by western blotting. As shown in Fig. 5a, the level of NF-κB p65 protein in the nucleus was decreased after treatment with 10 μM DYT-40 for 3, 6, 12, 18 and 24 h. Moreover, the total amount of NF-κB p65 decreased after DYT-40 treatment (Fig. 5b). Because NF-κB activation resulted from the rapid phosphorylation, ubiquitination and, ultimately, proteolytic degradation of IκB^33^ and because IKK is required for the phosphorylation of IκB^34^, the phosphorylation levels of IKKα/β and IκBα were detected. DYT-40 (12, 18 and 24 h) efficiently inhibited the phosphorylation of IKKα/β and IκBα, whereas the total steady-state levels remained unchanged (Fig. 5b).

For certification, pcDNA3.1 and pcDNA3.1-AEG-1 were transfected into U87 cells for 24 h, and the cells were treated with DYT-40 (10 μM) for an additional 24 h. The nuclei were isolated, and the amount of NF-κB p65 in the nuclear fraction was quantified using western blotting. As shown in Fig. 5c, the level of NF-κB p65 protein in the nucleus was evidently decreased after treatment with 10 μM DYT-40 for 24 h, and this effect was rescued by AEG-1 over-expression. The inhibition of both the distribution of AEG-1 and nuclear translocation of NF-κB p65 after DYT-40 (10 μM) treatment were observed using immunofluorescence confocal microscopy. This inhibition was rescued by AEG-1 over-expression (Fig. 5d).

As shown in Fig. 5e, DYT-40 treatment decreased the phosphorylation of AKT, and ERK kinase with no change in the total steady state protein levels. Furthermore, PI3K (p110) and PI3K (p85) was down-regulated and the tumor gene C-myc was down-regulated after treatment with DYT-40, suggesting that DYT-40 inhibited AEG-1 expression, which is involved in the suppression of the phosphorylation of PI3K/AKT and the MAPK axis. For certification, pcDNA3.1 and pcDNA3.1-AEG-1 were transfected into U87 cells for 24 h, and the cells were treated with DYT-40 (10 μM) for an additional 24 h. The inhibition of both the phosphorylation of AKT, and ERK kinase after DYT-40 (10 μM) treatment was rescued by AEG-1 over-expression (Fig. 5f). As shown in Fig. S4, DYT-40 inhibited the expression of AEG-1, C-myc, NF-κB p65, IκBα and p-IκBα in C6 cells in a concentration-dependent manner.

### DYT-40 reduced tumor volume and induced apoptotic cell death in the rat glioma model

**Figure 6:**
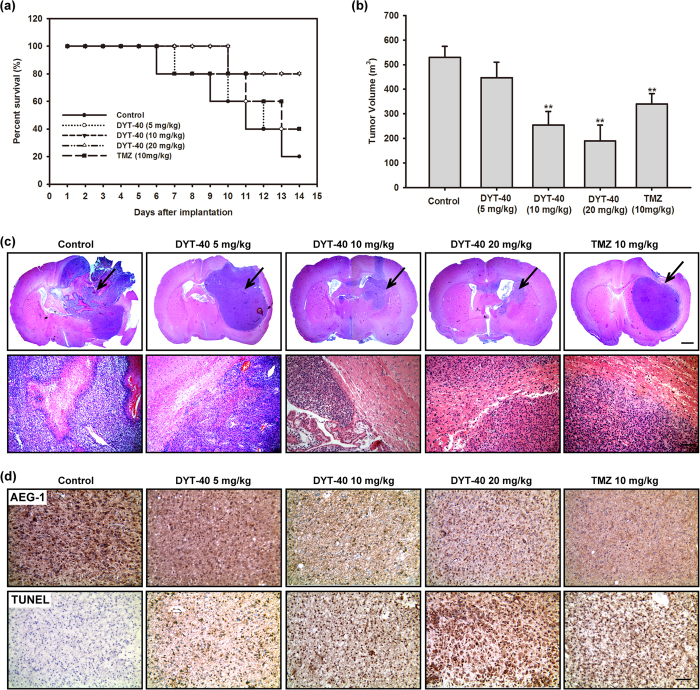
Administration of DYT-40 reduced tumor growth in the rat C6 glioma model. **(a)** The survival rate of the animals. **(b)** Tumor volumes in mm^3^ of DYT-40-treated and Temozolomide (TMZ)-treated animals compared with vehicle-treated animals. **(c)** A magnification of the striatum with a representative part of the tumor by H&E staining. DYT-40-treated animals showed smaller tumor volumes compared to untreated control animals (Scale bar: 1 mm, upper). The DYT-40-treated rats show less histopathological changes of the malignant C6 glioma compared to untreated animals (Scale bar: 100 μm, below). **(d)** Reduced AEG-1 expression and increased apoptosis caused by DYT-40 administration. Ten-micrometer thick cryostat sections are obtained and stained for AEG-1 and terminal deoxynucleotidyl transferase-mediated deoxyuridine triphosphate nick end labeling (TUNEL), and counterstained with hematoxylin.

To confirm the antineoplastic effects of DYT-40 for glioblastoma *in vivo*, C6 glioma cells were injected in rat striatum and treated with 5, 10, 20 mg/kg DYT-40 or vehicle for two weeks (n = 5 per group) at 24 h after initial tumor cell injection. On day 14, the rats in DYT-40-treated groups exhibited improved clinical outcomes and higher survival rates compared with the vehicle treated group (Fig. 6a). We did not find obvious pathological changes in rats after DYT-40 treatment. The DYT-40-treated animals showed a dramatic reduction in the tumor volume at 14 days of treatment compared with vehicle-treated animals (Fig. 6b). Vehicle-treated animals exhibited a median tumor volume of 530.08 mm^3^ ± 43.99, whereas 5, 10 and 20 mg/kg DYT-40-treated animals revealed tumor volumes of 447.38 mm^3^ ± 62.75, 254.61 mm^3^ ± 55.55, 190.08 mm^3^ ± 64.01, reflecting 16%, 52% and 64% reduction, respectively. Temozolomide treatment reduced tumor volumes 36% after 14 days (Fig. 6b).

Moreover, the results of H&E staining revealed that the glioma model rat treated with DYT-40 exhibited better defined tumor margins and fewer invasive cells to the contralateral striatum compared with vehicle control and temozolomide-treated rats (Fig. 6c). As shown in Fig. 6d, the microscopic examination of AEG-1-stained tumor sections shows a decrease in AEG-1-positive cells in DYT-40-treated rats compared with the untreated controls. The *in vivo* apoptotic response of glioma cells to DYT-40-treatment was investigated by TUNEL staining. The microscopic examination of the tumor sections showed that compared with the untreated controls, DYT-40-treatment increased the number of TUNEL-positive cells.

## Discussion

Since the discovery of AEG-1, many studies have focused on AEG-1 to expand the knowledge of this important molecule^35^. The crucial role of AEG-1 ranges from cancer biology to the molecular mechanisms underlying the biological functions. AEG-1 can be defined as a multifunctional oncogene that is over-expressed in complex types and progressive human cancers^6^,^10^,^11^,^36^. The elevated expression of AEG-1 in tumor cells enhanced the phenotypic characteristics of malignant aggressiveness, including increased robust proliferation, migration and invasion to surrounding tissues, neovascularization, and enhanced chemoresistance. The diverse functions of AEG-1 during tumor progression in various cancers including brain tumorigenesis have been elucidated. However, studies of AEG-1 inhibitors are urgently needed for clinical therapy. Recent studies have provided definitive evidence that elevated AEG-1 expression is a common event in brain tumors of diverse origin, including GBM^4^. In the present study, we focused on the regulatory mechanism of AEG-1/MTDH-mediated autophagy during malignant glioma EMT and invasion. An associated study has been published online in *Oncotarget*^37^. The results provided evidence that AEG-1 enhances human malignant glioma susceptibility to TGF-β1-triggered EMT via autophagy induction, which is associated with glioblastoma development and progression.

Several groups have demonstrated the diverse function of AEG-1 during the development and progression of cancers. The generalization of the signaling cascades associated with AEG-1 includes (1) the activation of the transcription factor nuclear factor κB (NF-κB), partially through direct interaction with p65, a TNF-α downstream signaling component, is associated with several human diseases, including cancer, and NF-κB controls the expression of multiple genes involved in tumor progression, invasion, and metastasis^8^; (2) enhancement of phosphorylation of MAPK molecules, including ERK1/2 and p38, which subsequently activates Wnt/β-catenin signaling and consequently results in tumor angiogenesis^10^; (3) suppressing apoptosis through a systematic mechanism that up-regulates apoptosis inhibitors via the indirect activation of the PI3K/AKT signaling pathway^19^; (4) recruitment of oncogene C-myc to the AEG-1 promoter region resulting from the activation of the Ras oncogene, which consequently activates AEG-1 expression^7^; (5) interaction with staphylococcal nuclease domain-containing 1 (SND1) and enhancing the activity of RNA-induced silencing complex (RISC), which leads to degradation of target of onco-miRNAs tumor suppressor mRNAs^38^; and (6) triggering protective autophagy, which is a common mechanism employed by tumor cells to adapt with metabolic stress^39^. In addition, we examined the mechanism that AEG-1/MTDH enhances human malignant glioma susceptibility to TGF-β1-triggered epithelial-mesenchymal transition via autophagy induction.

The inhibition of cancer cell proliferation by anticancer chemotherapeutic drugs is normally attributed to apoptosis, which is characterized by cytoplasmic shrinkage, chromatin condensation, and DNA fragmentation^40^. Apoptosis is an active form of programmed cell death that occurs in response to specific treatment, including mitochondria-mediated endogenous apoptosis and death receptor-mediated exogenous apoptosis^41^,^42^,^43^. As a consequence of adduct formation, DNA repair was simultaneously activated with cell cycle arrest. The failure to repair DNA led to cell apoptosis^44^. Mitochondria-mediated endogenous apoptosis is characterized by the decreased expression of apoptosis inhibitor proteins of the Bcl-2 gene family, including Bcl-2 and Mcl-1 and increases in the expression of the apoptotic protein Bax^45^,^46^. The involvement of caspase-8 and caspase-9 in apoptosis has been demonstrated by the cleavage of caspase-3 and PARP and the fragmentation of the caspase-3 substrate^46^,^47^. Thus, the members of both caspases and the Bcl-2 family as well as other proteins and events, such as oxidative stress and cell cycle arrest, could be defined as determinants of apoptosis^48^.

EMT is a complex biologic reprogramming that is considered a crucial event in metastasis and tumor progression^49^,^50^. At the early metastatic stage of tumors, the main feature of EMT occurrence is characterized by the loss of epithelial phenotype (E-cadherin) and acquisition of motility, invasive potential and mesenchymal characteristics (N-cadherin and vimentin)^51^. In addition, β-catenin, which originally combines with E-cadherin at the intracellular C-terminus, exhibits nuclear translocation and release from the membrane to promote a series of invasive molecules^52^. Simultaneously, AEG-1 activates Wnt/β-catenin signaling, which triggers EMT in some malignant tumors. As a result of the crucial role for the EMT in metastasis, suppressing the EMT checkpoint and progression in cancers has been recently considered a promising strategy to inhibit metastasis and prolong the survival of patients undergoing cancer.

The results of the present study have designed DYT-40, a novel synthetic (E)-2-(2-(4-chlorostyryl)-5-nitro-1H-imidazol-1-yl)ethyl-2-(2,3-dihydrobenzo[b][1,4]dioxin-6-yl) acetate, which significantly blocks malignant glioblastoma growth and invasion via the inhibition of AEG-1 expression. Moreover, the inhibition of AEG-1 expression significantly induces the apoptosis and suppresses the invasive ability of human malignant glioblastoma U251 and U87 cells by modulating MMP-2, MMP-9 activity and EMT characteristics. The activation and translocation of p65, the functional active subunit of NF-κB, were analyzed in U87 cells treated with DYT-40. The nuclei were isolated, and the amount of NF-κB p65 in the nuclear fraction was quantified by western blotting. The level of NF-κB p65 protein in the nucleus was evidently decreased after treatment with 10 μM DYT-40 for 3, 6, 12, 18, and 24 h. DYT-40 treatment decreased the phosphorylation of AKT and ERK kinase, with no change in the total steady state protein level. Furthermore, PI3K (p110) and PI3K (p85) and the tumor gene C-myc were down-regulated when treated with DYT-40, suggesting that DYT-40 inhibited AEG-1 expression, which is involved in the suppression of the phosphorylation of PI3K/AKT and MAPK.

In summary, these results indicated that DYT-40 inhibited the proliferation and invasion of malignant glioblastoma cells in a concentration-dependent manner. AEG-1 inhibition by DYT-40 was involved in the MAPK and PI3K/AKT-NF-κB signal transduction pathway and led to DYT-40-induced apoptosis and DYT-40-inhibited EMT in U251 and U87 cells. The inhibition of AEG-1 expression induced by DYT-40 resulted in the down-regulation of NF-κB expression, which exhibits a key molecular mechanism in which DYT-40 inhibits cell growth and invasion in malignant glioblastoma cells. To enter the brain, drugs must pass through the endothelial cells of the capillaries of the central nervous system (CNS) or be actively transported. Lipid-soluble drugs readily penetrate into the CNS because they can dissolve in the membrane of the endothelial cells (Page 554 of Lippincott’s Illustrated Reviews Pharmacology). DYT-40 is a synthetic derivative of nitroimidazole that can cross blood brain barrier because of its lipid-solubility. Accordingly, DYT-40 could penetrate the blood brain barrier following intravenous administration. In addition, DYT-40 with a low molecular weight of 455.85 has an enhanced ability to cross the blood-brain barrier even in the presence of meningeal inflammation. Actually, transport experiment of ^125^I-labeled DYT-40 on rat brain microvascular endothelial cells (rBMEC) would be more meaningful^53^.

Therefore, the inhibition of AEG-1 by the designed compound DYT-40 molecular targeting to AEG-1 might provide a novel strategy for the treatment of malignant glioblastoma cells. The results of the present study produced several important conclusions to further investigate the more potential role of AEG-1 in physiological or pathological processes and the sensitivity of cancer cells in response to clinically chemotherapeutic agents.

## Additional Information

**How to cite this article**: Zou, M. *et al*. DYT-40, a novel synthetic 2-styryl-5-nitroimidazole derivative, blocks malignant glioblastoma growth and invasion by inhibiting AEG-1 and NF-κB signaling pathways. *Sci. Rep.* **6**, 27331; doi: 10.1038/srep27331 (2016).

## Supplementary Material

Supplementary Information
