# Supplementary material for: DYT-40, a novel synthetic 2-styryl-5-nitroimidazole derivative, blocks malignant glioblastoma growth and invasion by inhibiting AEG-1 and NF-κB signaling pathways
Source: Sci Rep. 2016 Jun 2;6:27331. doi: 10.1038/srep27331 (PMC4890319; doi:10.1038/srep27331)
Supplement: Supplementary Information [file srep27331-s1.doc]

**DYT-40,** **a novel** **synthetic** **2-styryl-5-nitroimidazole derivative, blocks** **malignant** **glioblastoma growth and invasion by inhibiting AEG-1 and** **NF-κB signaling pathways**

Meijuan Zou^1^,^2^*, Yongtao Duan^2^, Pengfei Wang^2^, Rui Gao^1^, Xuguan Chen^1^, Yingwei Ou^1^, Mingxing Liang^1^, Zhongchang Wang^2^, Yi Yuan^3^, Li Wang^4^, Hailiang Zhu^1,2^*

^1^Department of Pharmacology, School of Basic Medical Sciences, Nanjing Medical University, Nanjing 210029, China; ^2^State Key Laboratory of Pharmaceutical Biotechnology, Nanjing University, Nanjing 210093, China; ^3^Jiangsu Key Laboratory of Oral Diseases; Department of oral and maxillofacial surgery, Affiliated Hospital of Stomatology, Nanjing Medical University, Nanjing 210029, China; ^4^Department of Breast Surgery, First Affiliated Hospital of Nanjing Medical University, Nanjing 210029, China.

To whom all the correspondence should be addressed at Department of Pharmacology, School of Basic Medical Sciences, Nanjing Medical University, Hanzhong Road 140^#^, Nanjing 210029, Jiangsu, China. Phone: 86-13813384465. E-mail: [zoumeijuan_njmu@163.com](mailto:zoumeijuan_njmu@163.com) (Meijuan Zou); [zhuhl@nju.edu.cn](mailto:zhuhl@nju.edu.cn) (Hailiang Zhu)

**Figure S1 The cell viability of MCF10A (human normal mammary epithelial cells) and HEK293T (human embryonic kidney 293T cells) after DYT-40 treatment.**


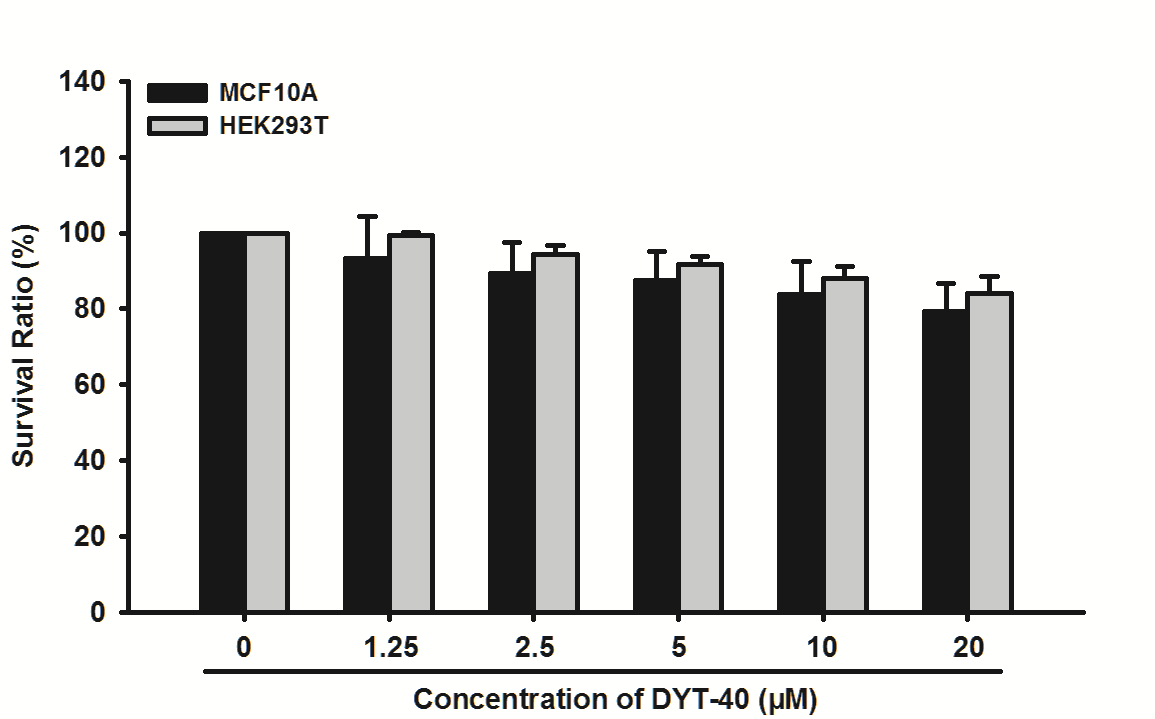


Figure S1 The cell viability of MCF10A (human normal mammary epithelial cells) and HEK293T (human embryonic kidney 293T cells) after DYT-40 treatment. Cells were treated with 0, 1.25, 2.5, 5, 10 and 20 μM DYT-40 for 48 h, respectively. Cell viability was determined by Cell Counting Kit-8 (CCK-8) assay.

**Figure S2 Temozolomide inhibited cell viability.**

**
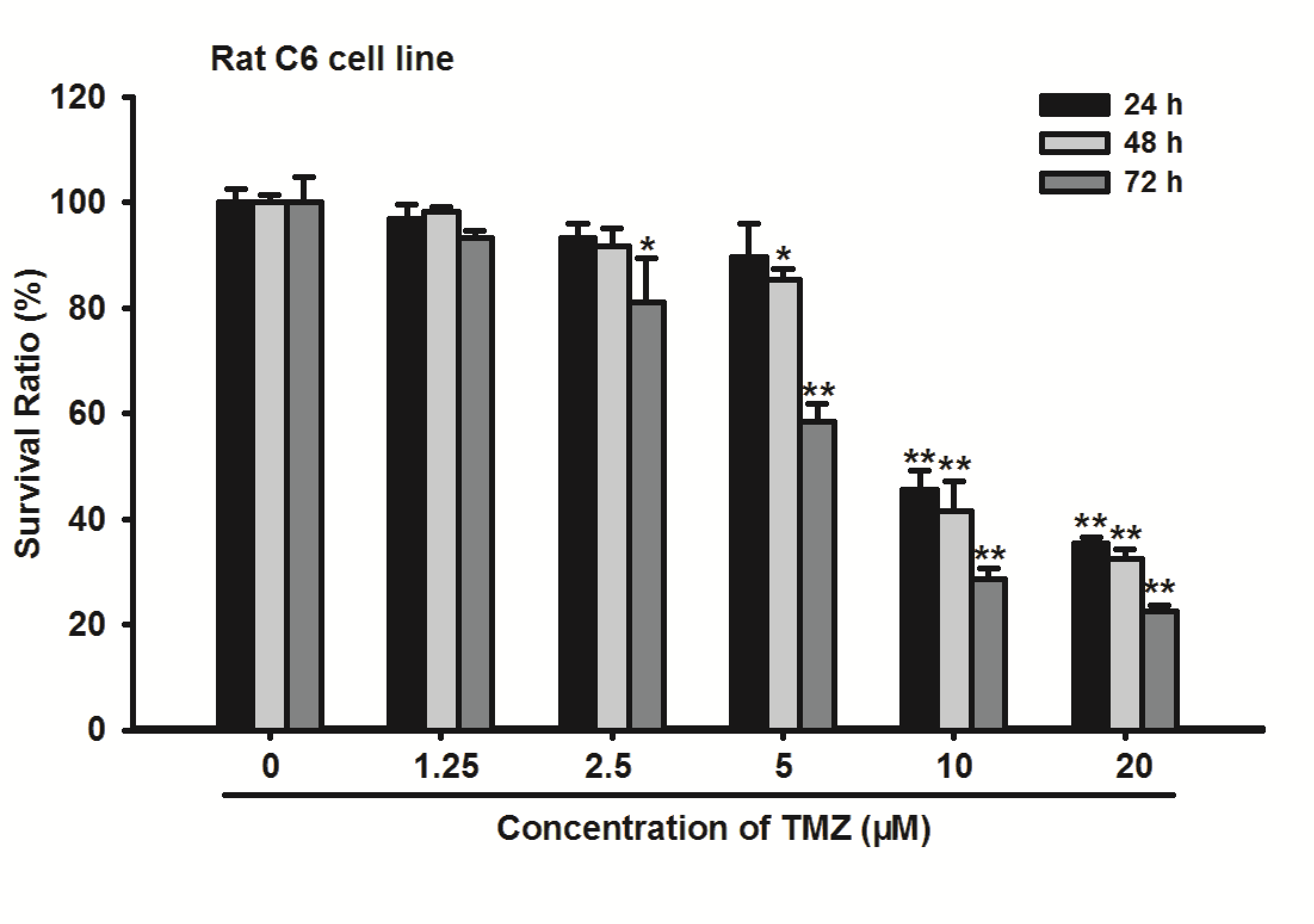
**

Figure S2 Temozolomide inhibited cell viability. Cell viability was determined by Cell Counting Kit-8 (CCK-8) assay. C6 cells were treated with 0, 1.25, 2.5, 5, 10 and 20 μM Temozolomide for 24 h, 48 h and 72 h, respectively. CCK-8 assay was used to measure cell viability.

**Figure S3 The cell viability and apoptosis of U251 and U87 cells after 3p treatment.**


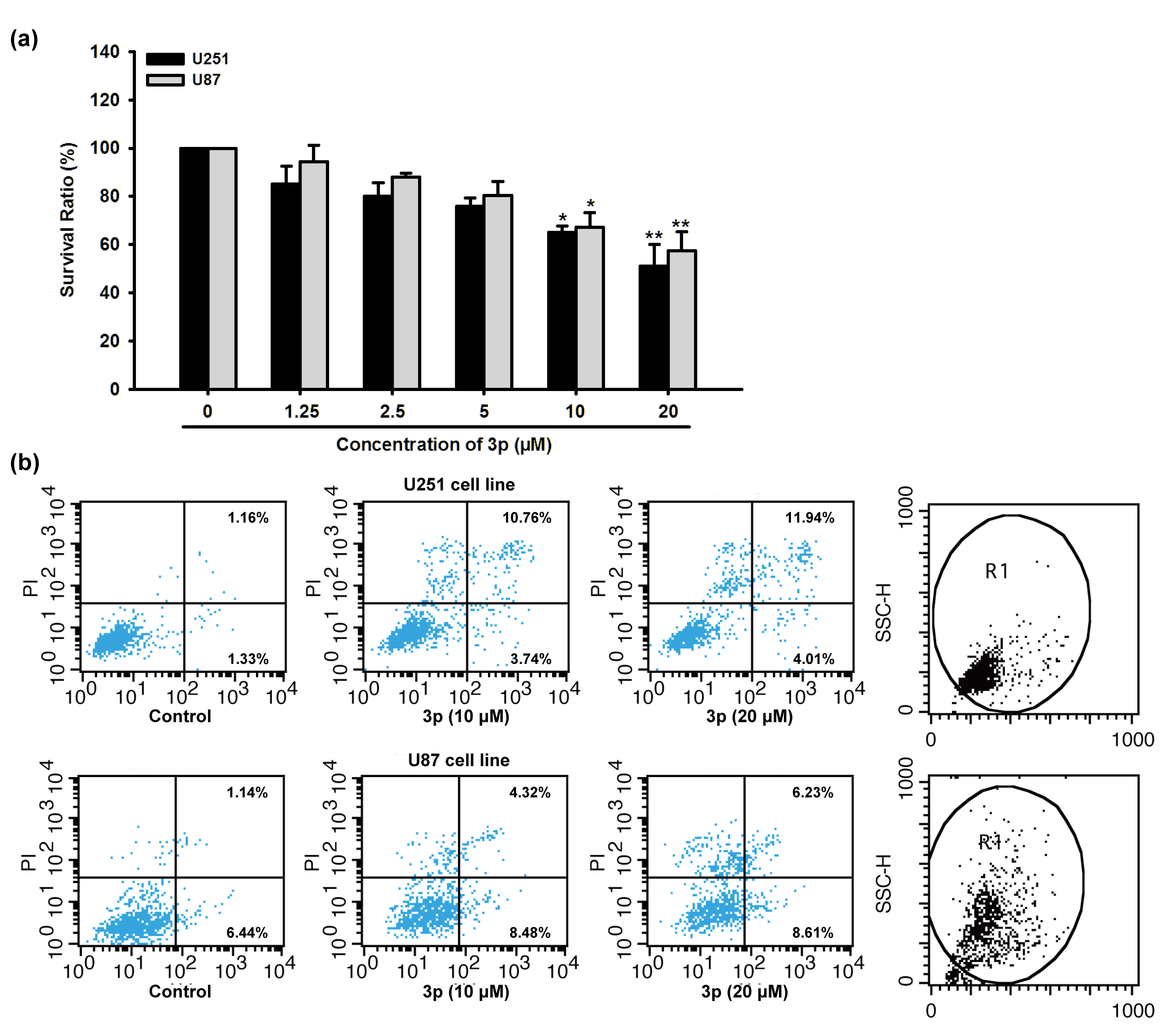


Figure S3 The cell viability and apoptosis of U251 and U87 cells after 3p treatment. (a) Cells were treated with 0, 1.25, 2.5, 5, 10 and 20 μM 3p for 72 h, respectively. Cell viability was determined by Cell Counting Kit-8 (CCK-8) assay. (b) Effect of 3p (10 and 20 μM) for 24 h on the apoptosis of cells was determined by Annexin V/PI staining assay.

**Figure S4 DYT-40 inhibited the expression of AEG-1, C-myc, NF-κB p65, IκBα and p-IκBα in C6 cells.**

**
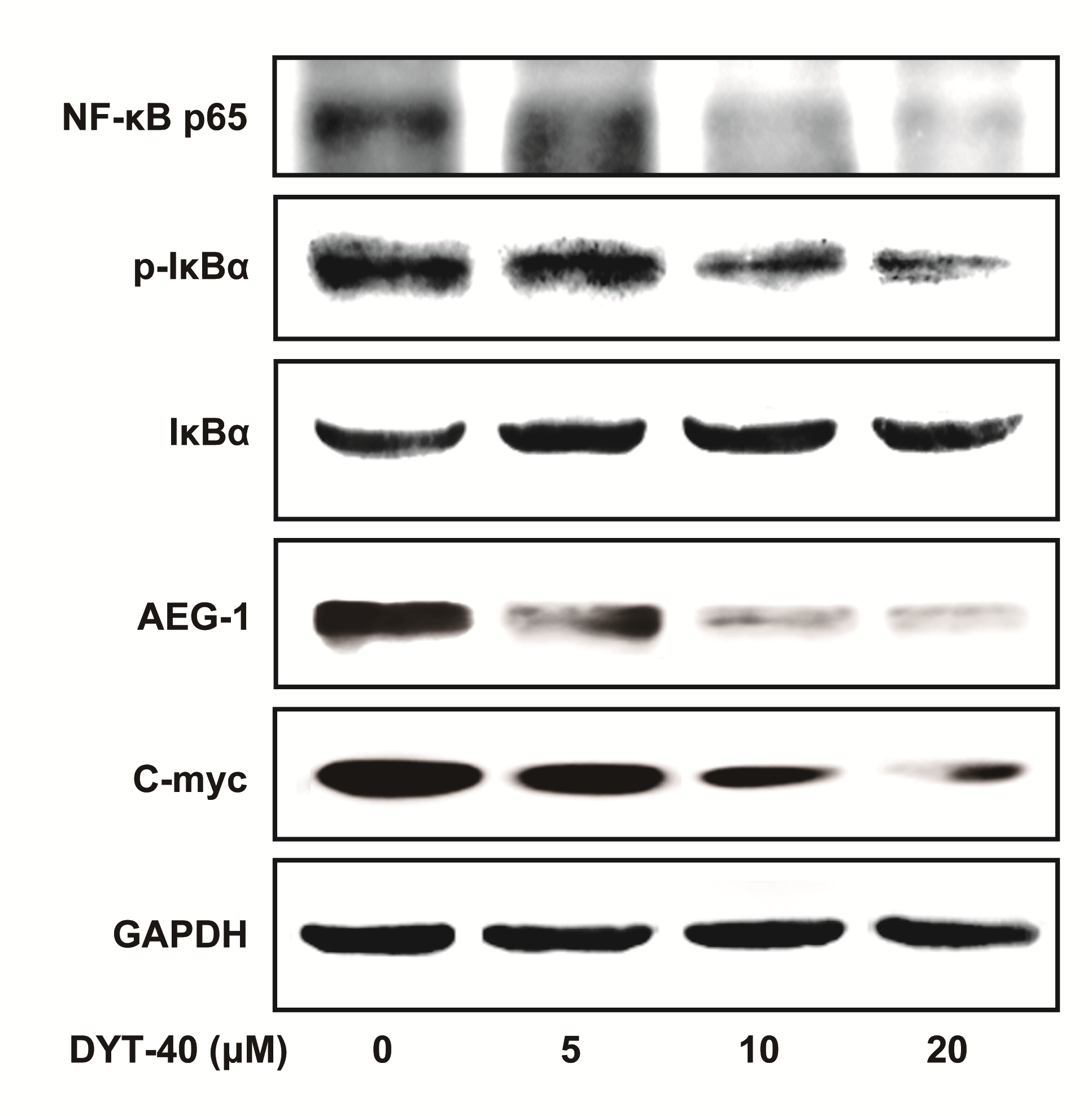
**

Figure S4 C6 cells were treated with the indicated concentrations of DYT-40 for 24 h. AEG-1, C-myc, NF-κB p65, IκBα and p-IκBα expression were evaluated by Western blotting.
